# Age and Gender Demographics Predict Compliance with COVID-19 Public Health Measures: Data from a Global Sample

**DOI:** 10.1093/geroni/igab046.3207

**Published:** 2021-12-17

**Authors:** Amber Heemskerk, Tian Lin, Elizabeth Harris, Jay Van Bavel, Natalie Ebner

**Affiliations:** 1 University of Florida, Gainesville, Florida, United States; 2 New York University, New York City, New York, United States; 3 New York University, New York, New York, United States

## Abstract

The COVID-19 global pandemic has brought far-reaching consequences on individual and societal levels. Social distancing and physical hygiene constitute effective public health measures to limit the spread of the virus. The current study investigates individual age and gender demographics, in interaction with a country’s human development index (HDI), as crucial factors influencing compliance with public health measures in a large multi-national adult lifespan sample. This report leverages data from a large-scale international collaboration (Van Bavel et al., 2020; https://psyarxiv.com/ydt95/) comprising 45,576 individuals from 66 countries/territories. Participants provided self-reports of their compliance/agreement with three public health measures (i.e., spatial distancing, physical hygiene, policy support). Older age, female gender, and lower HDI were independently associated with greater compliance with public health measures. In addition, a significant three-way interaction between participant age, participant gender, and a country’s HDI revealed that compliance was lowest in younger adults from well-developed countries, while compliance was highest among females across all ages from less-developed countries. Compliance with public health measures is crucial in effectively reducing coronavirus spread. Our findings suggest that age and gender as individual-level demographics, in tandem with HDI as a country-level predictor, affect individuals’ willingness to comply with public health measures. These results highlight the potential of data-driven, tailored (i.e., towards specific demographics) health campaigns and public policies in the fight against a global pandemic.

